# 2-[(4-Ethoxy­phen­yl)imino­meth­yl]-5-methoxy­phenol

**DOI:** 10.1107/S1600536809040240

**Published:** 2009-10-10

**Authors:** Zarife Sibel Şahin, Ferda Erşahin, Mustafa Macit, Şamil Işık

**Affiliations:** a Department of Physics, Faculty of Arts and Sciences, Ondokuz Mayıs University, Kurupelit, TR-55139 Samsun, Turkey; b Sinop University, Gerze Sinop Vocational School, Sinop, Turkey; c Department of Chemistry, Faculty of Arts and Sciences, Ondokuz Mayıs University, 55139 Samsun, Turkey

## Abstract

The title compound, C_16_H_17_NO_3_, a Schiff base, is stabilized in the solid state in the phenol–imine tautomeric form, with the H atom located on the hydr­oxy O atom rather than on the N atom. This H atom is involved in a strong intra­molecular O—H⋯N hydrogen bond. The mol­ecule is almost planar, the angle between the benzene rings being 4.43 (13)°. C—H⋯π inter­actions are also present.

## Related literature

For the industrial and biological properties of Schiff bases, see: Karia *et al.* (1999 ▶); Taggi *et al.* (2002 ▶). For Schiff base tautomerism, see: Şahin *et al.* (2005 ▶); Hadjoudis *et al.* (1987 ▶).
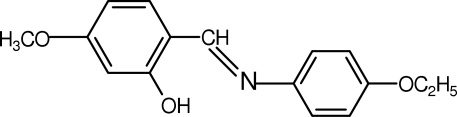


## Experimental

### 

#### Crystal data

C_16_H_17_NO_3_
*M* *_r_* = 271.31
Monoclinic, 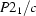

*a* = 7.4609 (7) Å
*b* = 8.3777 (5) Å
*c* = 23.016 (2) Å
β = 98.896 (8)°
*V* = 1421.3 (2) Å^3^
*Z* = 4
Mo *K*α radiation
μ = 0.09 mm^−1^
*T* = 296 K
0.49 × 0.28 × 0.07 mm

#### Data collection

Stoe IPDS-II diffractometer
Absorption correction: integration (*X-RED32*; Stoe & Cie, 2002 ▶) *T*_min_ = 0.983, *T*_max_ = 0.994
8971 measured reflections
2788 independent reflections
971 reflections with *I* > 2σ(*I*)
*R*_int_ = 0.068

#### Refinement

*R*[*F* ^2^ > 2σ(*F* ^2^)] = 0.042
*wR*(*F* ^2^) = 0.075
*S* = 0.80
2788 reflections
181 parameters
6 restraints
H-atom parameters constrained
Δρ_max_ = 0.06 e Å^−3^
Δρ_min_ = −0.09 e Å^−3^

### 

Data collection: *X-AREA* (Stoe & Cie, 2002 ▶); cell refinement: *X-AREA*; data reduction: *X-RED32* (Stoe & Cie, 2002 ▶); program(s) used to solve structure: *SHELXS97* (Sheldrick, 2008 ▶); program(s) used to refine structure: *SHELXL97* (Sheldrick, 2008 ▶); molecular graphics: *ORTEP-3 for Windows* (Farrugia, 1997 ▶); software used to prepare material for publication: *WinGX* (Farrugia, 1999 ▶).

## Supplementary Material

Crystal structure: contains datablocks I, global. DOI: 10.1107/S1600536809040240/bh2250sup1.cif

Structure factors: contains datablocks I. DOI: 10.1107/S1600536809040240/bh2250Isup2.hkl

Additional supplementary materials:  crystallographic information; 3D view; checkCIF report

## Figures and Tables

**Table 1:** Hydrogen-bond geometry (Å, °)

*D*—H⋯*A*	*D*—H	H⋯*A*	*D*⋯*A*	*D*—H⋯*A*
O1—H1⋯N1	0.82	1.83	2.563 (3)	148
C1—H1*A*⋯*Cg*2^i^	0.96	3.32	4.000 (3)	129
C3—H3⋯*Cg*2^i^	0.93	3.30	4.155 (3)	155
C8—H8⋯*Cg*2^ii^	0.93	3.39	4.269 (3)	159
C10—H10⋯*Cg*1^i^	0.93	3.00	3.849 (3)	153
C16—H16*B*⋯*Cg*1^ii^	0.97	3.04	3.822 (3)	139

Symmetry codes: (i) 
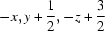
; (ii) 
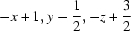
. *Cg*1 and *Cg*2 are the centroids of the [please define] and [please define] rings, respectively.

**Fig. 1.**
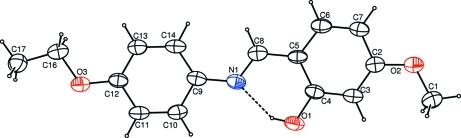
The molecular structure of the title compound, showing the atom-numbering scheme. Displacement ellipsoids are drawn at the 30% probability level.

**Fig. 2.**
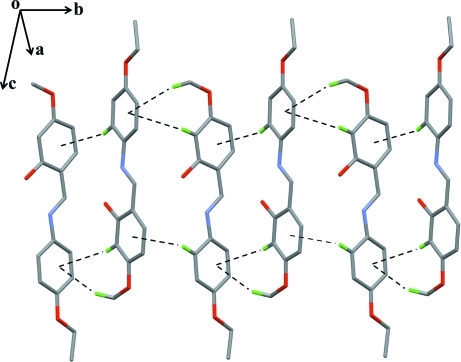
Part of the crystal structure of the title compound, showing the formation of a chain along [010] generated by the C—H···π interactions.

## supplementary crystallographic information

### Comment

Schiff bases are used as substrates in the preparation of a number of industrial and biologically active compounds *via* ring closure, cycloaddition and replacement reactions (Karia *et al.*, 1999). Moreover, Schiff bases are also known to have biological activities such as antimicrobial, antifungal, antitumor and as herbicides. On the industrial scale, they have a wide range of applications such as dyes and pigments (Taggi *et al.*, 2002). In general, Schiff bases display two possible tautomeric forms, the phenol-imine (Hadjoudis *et al.*, 1987) and keto-amine (Şahin *et al.*, 2005) forms. Depending on the tautomers two types of intramolecular hydrogen bonds are observed in Schiff bases: O—H···N in phenol-imine and N—H···O in keto-amine tautomers. Our X-ray investigation of the title compound indicates that the phenol-imine tautomer is favoured over the keto-amine tautomer (Fig. 1).

Selected bond lengths of the title compound are given in Table 1. The N1—C8 bond length of 1.285 (3) Å is typical of a double bond. The dihedral angle between the C2···C7 and C9···C14 benzene rings is 4.43 (13)° and the compound is thus almost planar. The C5—C8—N1—C9 torsion angle is 179.3 (2)°. The compound shows a strong intramolecular hydrogen bond (O1—H1···N1) which forms a *S*(6) motif. The compound also contains five intermolecular C—H···π interactions. The combination of three C—H···π interactions generates chain of edge-fused *R*_2_^1^(7)*R*_2_^2^(15) rings running parallel to the [010] direction (Fig. 2). The details of C—H···π interactions are given in Table 2.

### Experimental

The title compound was prepared by refluxing a mixture of a solution containing 2-hydroxy-4-methoxy-benzaldehyde (0.0237 g, 0.156 mmol) in 20 ml ethanol and a solution containing 4-ethoxyaniline (0.0214 g, 0.156 mmol) in 20 ml ethanol. The reaction mixture was stirred for 1 h under reflux. Crystals suitable for X-ray analysis were obtained from ethylalcohol by slow evaporation (yield % 71; m.p. 381–383 K).

### Refinement

Phenolic H atom (H1) was first detected in a difference map, but eventually fixed in calculated position, with the O—H bond length constrained to 0.82 Å and *U*_iso_(H1) = 1.5*U*_eq_(O1). Other H atoms were also placed in calculated positions and constrained to ride on their parents atoms, with C—H = 0.93–0.96 Å, and *U*_iso_(H) = 1.2*U*_eq_(C) or 1.5*U*_eq_(C). For atom C12, anisotropic displacement parameters were restrained to approximate an isotropic behaviour.

### Crystal data

**Table:**

C_16_H_17_NO_3_	*F*(000) = 576
*M**_r_* = 271.31	*D*_x_ = 1.268 Mg m^−^^3^
Monoclinic, *P*2_1_/*c*	Melting point: 381 K
Hall symbol: -P 2ybc	Mo *K*α radiation, λ = 0.71073 Å
*a* = 7.4609 (7) Å	Cell parameters from 5881 reflections
*b* = 8.3777 (5) Å	θ = 1.8–27.3°
*c* = 23.016 (2) Å	µ = 0.09 mm^−^^1^
β = 98.896 (8)°	*T* = 296 K
*V* = 1421.3 (2) Å^3^	Plate, yellow
*Z* = 4	0.49 × 0.28 × 0.07 mm

### Data collection

**Table:**

Stoe IPDS-II diffractometer	2788 independent reflections
Radiation source: fine-focus sealed tube	971 reflections with *I* > 2σ(*I*)
graphite	*R*_int_ = 0.068
Detector resolution: 6.67 pixels mm^-1^	θ_max_ = 26.0°, θ_min_ = 1.8°
ω scans	*h* = −8→9
Absorption correction: integration (*X-RED32*; Stoe & Cie, 2002)	*k* = −8→10
*T*_min_ = 0.983, *T*_max_ = 0.994	*l* = −28→28
8971 measured reflections	

### Refinement

**Table:**

Refinement on *F*^2^	Primary atom site location: structure-invariant direct methods
Least-squares matrix: full	Secondary atom site location: difference Fourier map
*R*[*F*^2^ > 2σ(*F*^2^)] = 0.042	Hydrogen site location: inferred from neighbouring sites
*wR*(*F*^2^) = 0.075	H-atom parameters constrained
*S* = 0.80	*w* = 1/[σ^2^(*F*_o_^2^) + (0.0187*P*)^2^] where *P* = (*F*_o_^2^ + 2*F*_c_^2^)/3
2788 reflections	(Δ/σ)_max_ < 0.001
181 parameters	Δρ_max_ = 0.06 e Å^−^^3^
6 restraints	Δρ_min_ = −0.09 e Å^−^^3^
0 constraints	

### Fractional atomic coordinates and isotropic or equivalent isotropic displacement parameters (Å^2^)

**Table:**

	*x*	*y*	*z*	*U*_iso_*/*U*_eq_	
C1	−0.6165 (4)	0.5043 (3)	0.57051 (10)	0.1085 (9)	
H1A	−0.5696	0.6108	0.5697	0.163*	
H1B	−0.6483	0.4828	0.6086	0.163*	
H1C	−0.7222	0.4938	0.5411	0.163*	
C2	−0.3202 (4)	0.3896 (3)	0.59654 (12)	0.0808 (7)	
C3	−0.2831 (4)	0.4771 (3)	0.64744 (12)	0.0856 (8)	
H3	−0.3693	0.5465	0.6584	0.103*	
C4	−0.1155 (5)	0.4605 (3)	0.68216 (11)	0.0800 (7)	
C5	0.0154 (4)	0.3552 (3)	0.66750 (11)	0.0757 (7)	
C6	−0.0289 (4)	0.2690 (3)	0.61528 (12)	0.0893 (8)	
H6	0.0557	0.1980	0.6044	0.107*	
C7	−0.1921 (4)	0.2855 (3)	0.57975 (11)	0.0870 (8)	
H7	−0.2173	0.2281	0.5449	0.104*	
C8	0.1875 (4)	0.3370 (3)	0.70488 (12)	0.0838 (8)	
H8	0.2693	0.2612	0.6953	0.101*	
C9	0.3994 (5)	0.4049 (3)	0.78873 (12)	0.0795 (7)	
C10	0.4158 (4)	0.4914 (3)	0.84077 (13)	0.0890 (8)	
H10	0.3202	0.5556	0.8483	0.107*	
C11	0.5712 (5)	0.4833 (3)	0.88123 (12)	0.0910 (8)	
H11	0.5793	0.5423	0.9158	0.109*	
C12	0.7163 (4)	0.3890 (3)	0.87168 (12)	0.0808 (7)	
C13	0.7025 (4)	0.3066 (3)	0.81961 (12)	0.0921 (8)	
H13	0.7997	0.2452	0.8117	0.111*	
C14	0.5468 (5)	0.3141 (3)	0.77915 (12)	0.0936 (8)	
H14	0.5403	0.2564	0.7444	0.112*	
O3	0.8627 (3)	0.38748 (19)	0.91485 (8)	0.0937 (5)	
C16	1.0174 (4)	0.2954 (3)	0.90641 (11)	0.0995 (8)	
H16A	1.0645	0.3324	0.8718	0.119*	
H16B	0.9841	0.1839	0.9008	0.119*	
C17	1.1580 (4)	0.3138 (3)	0.95957 (11)	0.1148 (10)	
H17A	1.2637	0.2532	0.9546	0.172*	
H17B	1.1108	0.2758	0.9935	0.172*	
H17C	1.1900	0.4245	0.9649	0.172*	
N1	0.2308 (3)	0.4230 (2)	0.75114 (10)	0.0830 (6)	
O1	−0.0840 (2)	0.55210 (18)	0.73075 (7)	0.1034 (6)	
H1	0.0175	0.5328	0.7486	0.155*	
O2	−0.4817 (3)	0.39332 (19)	0.55872 (7)	0.0959 (5)	

### Atomic displacement parameters (Å^2^)

**Table:**

	*U*^11^	*U*^22^	*U*^33^	*U*^12^	*U*^13^	*U*^23^
C1	0.104 (2)	0.111 (2)	0.119 (2)	0.008 (2)	0.0432 (17)	−0.0031 (16)
C2	0.101 (3)	0.0615 (16)	0.086 (2)	0.0006 (18)	0.0332 (17)	0.0117 (15)
C3	0.113 (3)	0.0661 (17)	0.0850 (18)	−0.0012 (17)	0.0390 (17)	−0.0099 (15)
C4	0.110 (2)	0.0633 (16)	0.0734 (18)	−0.0098 (18)	0.0350 (17)	−0.0052 (14)
C5	0.107 (3)	0.0510 (15)	0.0770 (18)	0.0034 (16)	0.0393 (17)	0.0008 (13)
C6	0.128 (3)	0.0612 (16)	0.0856 (19)	0.0098 (17)	0.0385 (17)	0.0017 (15)
C7	0.122 (3)	0.0655 (17)	0.0777 (18)	0.0055 (18)	0.0277 (18)	−0.0040 (13)
C8	0.110 (3)	0.0594 (16)	0.0882 (18)	0.0026 (16)	0.0367 (17)	0.0105 (15)
C9	0.113 (3)	0.0539 (16)	0.0788 (19)	−0.0098 (17)	0.0378 (18)	−0.0004 (14)
C10	0.095 (2)	0.0694 (17)	0.109 (2)	−0.0008 (16)	0.0387 (17)	−0.0109 (16)
C11	0.106 (2)	0.0722 (18)	0.103 (2)	−0.0002 (19)	0.0425 (18)	−0.0223 (15)
C12	0.104 (3)	0.0599 (16)	0.0849 (19)	−0.0066 (17)	0.0352 (18)	−0.0046 (14)
C13	0.107 (3)	0.0849 (19)	0.090 (2)	0.0093 (18)	0.0337 (17)	−0.0042 (16)
C14	0.126 (3)	0.0754 (18)	0.089 (2)	0.009 (2)	0.044 (2)	−0.0130 (15)
O3	0.0995 (16)	0.0828 (12)	0.1030 (13)	0.0054 (11)	0.0288 (12)	−0.0143 (9)
C16	0.106 (3)	0.0844 (18)	0.117 (2)	0.0038 (18)	0.0463 (19)	−0.0041 (15)
C17	0.120 (3)	0.118 (2)	0.107 (2)	0.0166 (19)	0.0188 (19)	0.0062 (16)
N1	0.111 (2)	0.0614 (13)	0.0824 (16)	−0.0081 (13)	0.0317 (14)	−0.0039 (11)
O1	0.1215 (16)	0.0934 (12)	0.0997 (12)	0.0025 (11)	0.0312 (10)	−0.0327 (10)
O2	0.1132 (17)	0.0798 (12)	0.0985 (13)	0.0076 (12)	0.0283 (12)	−0.0057 (9)

### Geometric parameters (Å, °)

**Table:**

C1—O2	1.426 (3)	C9—C10	1.389 (3)
C1—H1A	0.9600	C9—N1	1.420 (3)
C1—H1B	0.9600	C10—C11	1.372 (3)
C1—H1C	0.9600	C10—H10	0.9300
C2—O2	1.374 (3)	C11—C12	1.385 (3)
C2—C3	1.374 (3)	C11—H11	0.9300
C2—C7	1.392 (3)	C12—O3	1.358 (3)
C3—C4	1.383 (3)	C12—C13	1.373 (3)
C3—H3	0.9300	C13—C14	1.373 (3)
C4—O1	1.347 (2)	C13—H13	0.9300
C4—C5	1.396 (3)	C14—H14	0.9300
C5—C6	1.398 (3)	O3—C16	1.426 (3)
C5—C8	1.439 (3)	C16—C17	1.492 (3)
C6—C7	1.364 (3)	C16—H16A	0.9700
C6—H6	0.9300	C16—H16B	0.9700
C7—H7	0.9300	C17—H17A	0.9600
C8—N1	1.285 (3)	C17—H17B	0.9600
C8—H8	0.9300	C17—H17C	0.9600
C9—C14	1.383 (3)	O1—H1	0.8200
			
O2—C1—H1A	109.5	C11—C10—H10	119.6
O2—C1—H1B	109.5	C9—C10—H10	119.6
H1A—C1—H1B	109.5	C10—C11—C12	121.3 (3)
O2—C1—H1C	109.5	C10—C11—H11	119.3
H1A—C1—H1C	109.5	C12—C11—H11	119.3
H1B—C1—H1C	109.5	O3—C12—C13	125.3 (3)
O2—C2—C3	124.7 (3)	O3—C12—C11	116.7 (3)
O2—C2—C7	114.3 (3)	C13—C12—C11	118.1 (3)
C3—C2—C7	121.0 (3)	C12—C13—C14	120.7 (3)
C2—C3—C4	118.9 (3)	C12—C13—H13	119.6
C2—C3—H3	120.5	C14—C13—H13	119.6
C4—C3—H3	120.5	C13—C14—C9	121.8 (3)
O1—C4—C3	116.6 (3)	C13—C14—H14	119.1
O1—C4—C5	121.5 (3)	C9—C14—H14	119.1
C3—C4—C5	121.9 (3)	C12—O3—C16	118.8 (2)
C4—C5—C6	116.9 (3)	O3—C16—C17	108.3 (2)
C4—C5—C8	121.1 (3)	O3—C16—H16A	110.0
C6—C5—C8	122.0 (3)	C17—C16—H16A	110.0
C7—C6—C5	122.3 (3)	O3—C16—H16B	110.0
C7—C6—H6	118.9	C17—C16—H16B	110.0
C5—C6—H6	118.9	H16A—C16—H16B	108.4
C6—C7—C2	119.0 (3)	C16—C17—H17A	109.5
C6—C7—H7	120.5	C16—C17—H17B	109.5
C2—C7—H7	120.5	H17A—C17—H17B	109.5
N1—C8—C5	121.5 (3)	C16—C17—H17C	109.5
N1—C8—H8	119.2	H17A—C17—H17C	109.5
C5—C8—H8	119.2	H17B—C17—H17C	109.5
C14—C9—C10	117.3 (3)	C8—N1—C9	122.1 (2)
C14—C9—N1	127.8 (3)	C4—O1—H1	109.5
C10—C9—N1	114.9 (3)	C2—O2—C1	118.0 (2)
C11—C10—C9	120.8 (3)		
			
O2—C2—C3—C4	179.0 (2)	C9—C10—C11—C12	−0.1 (4)
C7—C2—C3—C4	0.0 (3)	C10—C11—C12—O3	−179.1 (2)
C2—C3—C4—O1	178.26 (19)	C10—C11—C12—C13	1.8 (4)
C2—C3—C4—C5	−1.2 (3)	O3—C12—C13—C14	178.9 (2)
O1—C4—C5—C6	−178.2 (2)	C11—C12—C13—C14	−2.0 (4)
C3—C4—C5—C6	1.2 (3)	C12—C13—C14—C9	0.6 (4)
O1—C4—C5—C8	1.9 (3)	C10—C9—C14—C13	1.1 (3)
C3—C4—C5—C8	−178.6 (2)	N1—C9—C14—C13	−179.7 (2)
C4—C5—C6—C7	0.0 (3)	C13—C12—O3—C16	0.7 (3)
C8—C5—C6—C7	179.8 (2)	C11—C12—O3—C16	−178.4 (2)
C5—C6—C7—C2	−1.2 (4)	C12—O3—C16—C17	179.9 (2)
O2—C2—C7—C6	−178.0 (2)	C5—C8—N1—C9	179.3 (2)
C3—C2—C7—C6	1.2 (3)	C14—C9—N1—C8	8.9 (3)
C4—C5—C8—N1	−4.0 (3)	C10—C9—N1—C8	−171.8 (2)
C6—C5—C8—N1	176.2 (2)	C3—C2—O2—C1	4.8 (3)
C14—C9—C10—C11	−1.3 (3)	C7—C2—O2—C1	−176.0 (2)
N1—C9—C10—C11	179.3 (2)		

### Hydrogen-bond geometry (Å, °)

**Table:**

*D*—H···*A*	*D*—H	H···*A*	*D*···*A*	*D*—H···*A*
O1—H1···N1	0.82	1.83	2.563 (3)	148
C1—H1A···Cg2^i^	0.96	3.32	4.000 (3)	129
C3—H3···Cg2^i^	0.93	3.30	4.155 (3)	155
C8—H8···Cg2^ii^	0.93	3.39	4.269 (3)	159
C10—H10···Cg1^i^	0.93	3.00	3.849 (3)	153
C16—H16B···Cg1^ii^	0.97	3.04	3.822 (3)	139

Symmetry codes: (i) −*x*, *y*+1/2, −*z*+3/2; (ii) −*x*+1, *y*−1/2, −*z*+3/2.
